# miR-23a suppresses pancreatic cancer cell progression by inhibiting PLK-1 expression

**DOI:** 10.3892/mmr.2022.12738

**Published:** 2022-05-17

**Authors:** Bin Chen, Akao Zhu, Lei Tian, Ying Xin, Xinchun Liu, Yunpeng Peng, Jingjing Zhang, Yi Miao, Jishu Wei

Mol Med Rep 18: 105–112, 2018; DOI: 10.3892/mmr.2018.8941

Subsequently to the publication of this paper, the authors have realized that they made an error during the sorting of the data panels shown for the migration and invasion assays shown in Fig. 2C; essentially, the ‘Invasion/PLL3.7’ panel was chosen from the same original data source as the panel selected to represent the ‘Migration/Inhibitor-NC’ experiment.

The authors have consulted their original data, and realize that the ‘Invasion/PLL3.7’ data panel was inadvertently selected incorrectly for Fig. 2. The revised version of Fig. 2, showing the data appropriate for the ‘Invasion/PLL3.7’ experiment, is shown on the next page. Note that the errors made in assembling Fig. 2 did not significantly affect either the results or the conclusions reported in this paper, and all the authors agree to this Corrigendum. The authors are grateful to the Editor of *Molecular Medicine Reports* for allowing them the opportunity to publish this corrigendum, and apologize to the readership for any inconvenience caused.

## Figures and Tables

**Figure 2.**
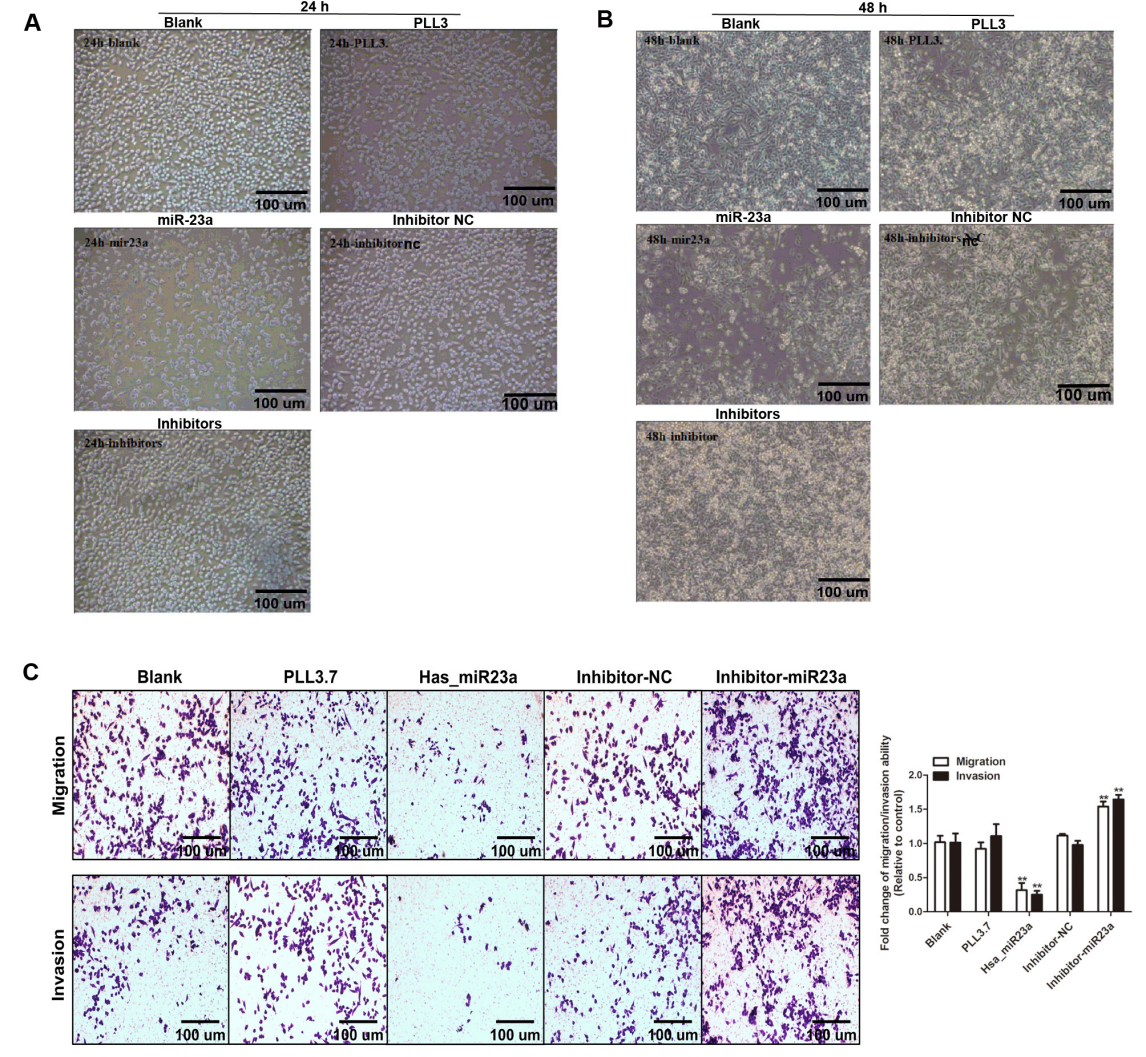
Effects of miR-23a on proliferation and metastasis ability of pancreatic cancer cells. Cell proliferation ability was examined in the Blank, inhibitor-NC, PLL 3.7, miR-23a and inhibitor groups after (A) 24 h and (B) 48 h treatment. (C) The migration and invasion cell number was stained with crystal violet and numbered in each group. Data were presented as the mean ± standard deviation. **P<0.01 vs. the blank group. NC, negative control; miR, microRNA.

